# A Prospective, Observational Study of the Effect of a High-Calorie, High-Protein Oral Nutritional Supplement with HMB in an Old and Malnourished or at-Risk-of-Malnutrition Population with Hip Fractures: A FracNut Study

**DOI:** 10.3390/nu16081223

**Published:** 2024-04-19

**Authors:** Teresa Pareja Sierra, Flavia Lorena Hünicken Torrez, María Carmen Pablos Hernández, Rosario López Velasco, Raquel Ortés Gómez, María del Carmen Cervera Díaz, Ana Isabel Hormigo Sánchez, Beatriz Perdomo Ramírez, Jesús Mora Fernández, Sonia Jiménez Mola, María Amparo Rodriguez Piñera, Patricia Ysabel Condorhuaman Alvarado, Carlos Sanchez Juan, Juan Ignacio Ramos Clemente, Silvia Veses Martín, Ingrid Rodríguez Manzano, Magali González-Colaço Harmand, María Camprubí Robles, Andrea Martín Aguilar, Pilar Saez Lopez

**Affiliations:** 1 Department of Geriatrics, University Hospital of Guadalajara, 19002 Guadalajara, Spain; tparejas2@hotmail.com; 2 Department of Geriatrics, Healthcare Complex of Ávila, 05071 Ávila, Spain; flhunicken@yahoo.com.ar; 3 Department of Geriatrics, University Hospital of Salamanca, 37007 Salamanca, Spain; 4 Department of Geriatrics, University Hospital Nuestra Señora de Valme, 41014 Sevilla, Spain; 5 Department of Geriatrics, University Hospital San Pedro de Alcántara, 10003 Cáceres, Spain; 6 Department of Geriatrics, University Clinical Hospital of Valladolid, 47007 Valladolid, Spain; mcerveradiaz@gmail.com; 7 Department of Geriatrics, University Hospital Fundación Jiménez Díaz, 28040 Madrid, Spain; 8 Department of Geriatrics, University Hospital Fundación Alcorcón, 28922 Alcorcón, Spainpisalop@gmail.com (P.S.L.); 9 Department of Geriatrics, Instituto de Investigación del Hospital Clínico San Carlos (IdISSC), Universidad Complutense, 28040 Madrid, Spain; 10 Department of Geriatrics, Complejo Asistencial Universitario de León, 24008 León, Spain; 11 Department of Geriatrics, Cruz Roja Gijón Hospital, 33202 Gijón, Spain; 12 La Paz Hospital Research Institute (IdiPAZ), 28029 Madrid, Spain; 13 Geriatrics Department, University Hospital La Paz, 28046 Madrid, Spain; 14 Department of Endocrinology and Nutrition, Hospital General University of Valencia, 46014 València, Spain; 15 Internal Medicine, Infanta Elena Hospital, 28342 Valdemoro, Spain; 16 Departament of Endocrinology, Doctor Peset University Hospital, 46017 València, Spain; 17 Departament of Geriatrics, University Hospital Gran Canaria Doctor Negrín, 35010 Las Palmas de Gran Canaria, Spain; 18 Departament of Geriatrics, University Hospital Nuestra Señora de Candelaria, 38010 Santa Cruz de Tenerife, Spain; magaligch@hotmail.com; 19 Abbott Nutrition R&D, 18004 Granada, Spain; maria.camprubirobles@abbott.com; 20 Medical Advisor, Abbott Nutrition, 28050 Madrid, Spain; 21 Head Coordinator of the Spanish National Hip Fracture Registry, Madrid, Spain

**Keywords:** malnutrition, hip fracture, ONS-beta-hydroxy-beta-methylbutyrate

## Abstract

Background: Hip fractures are prevalent among older people, often leading to reduced mobility, muscle loss, and bone density decline. Malnutrition exacerbates the prognosis post surgery. This study aimed to evaluate the impact of a 12-week regimen of a high-calorie, high-protein oral supplement with β-hydroxy-β-methylbutyrate (HC-HP-HMB-ONS) on nutritional status, daily activities, and compliance in malnourished or at-risk older patients with hip fractures receiving standard care. Subjects and Methods: A total of 270 subjects ≥75 years of age, residing at home or in nursing homes, malnourished or at risk of malnutrition, and post hip fracture surgery, received HC-HP-HMB-ONS for 12 weeks. Various scales and questionnaires assessed outcomes. Results: During the 12 weeks of follow-up, 82.8% consumed ≥75% of HC-HP-HMB-ONS. By week 12, 62.4% gained or maintained weight (+0.3 kg), 29.2% achieved normal nutritional status (mean MNA score +2.8), and 46.8% improved nutritional status. Biochemical parameters improved significantly. Subjects reported good tolerability (mean score 8.5/10), with 87.1% of healthcare providers concurring. Conclusions: The administration of HC-HP-HMB-ONS markedly enhanced nutritional status and biochemical parameters in older hip-fracture patients, with high compliance and tolerability. Both patients and healthcare professionals expressed satisfaction with HC-HP-HMB-ONS.

## 1. Introduction

Hip fractures are a major public health problem, associated with increased care needs and high morbidity and mortality, especially in older people [1]. The incidence of hip fractures varies widely among countries [2]. In Spain, the hip-fracture incidence rate is estimated at 100 per 100,000 inhabitants [3].

Hip fractures are common in older adults, and the predominant mechanism of injury is low-energy trauma (i.e., a fall from a standing height). Subjects with hip fractures usually require early surgery and a multidisciplinary approach [4].

The most important factors in the pathogenesis of hip fractures are those that negatively affect bone mineral density such as advanced age, female sex, previous fractures, systemic disease, medication, lifestyle factors related to physical activity, functional disability, and malnutrition [5,6]. In addition, the loss of muscle mass, strength, and function associated with ageing increases the risk of falls and, subsequently, the risk of fractures [7]. On the other hand, dietary factors represent modifiable risk factors that can improve bone and muscle health, decreasing the risk of hip fracture and accelerating people’s recovery after a hip fracture [5].

The presence of malnutrition or risk of malnutrition at the time of a hip fracture is a factor that is also related to an increase in the length of hospital stay after intervention, as well as an increased risk of complications, morbidity, and mortality [6]. Thus, the European Society for Clinical Nutrition and Metabolism bases its recommendations on the evidence behind the use of nutritional supplements with specific nutrients, such as β-hydroxy-β-methylbutyrate (HMB) and vitamin D in polymorbid subjects [8].

HMB has been shown to improve synthesis and reduce the degradation of muscle proteins [9]. Endogenous production of HMB decreases with age, and low levels are associated with a loss of appendicular lean mass and grip strength [10]. Supplementation with HMB prevents muscle loss associated with bed confinement by stimulating the mTOR target [10,11].

Vitamin D plays an important role in protein synthesis. Vitamin D deficiency is very common among older adults and postmenopausal women [12,13], and it is associated with decreased muscle strength and an increased risk of developing mobility limitations and disability [14,15]. Vitamin D supplements may help improve muscle function as well as strength and reduce the risk of falls [16] and mortality [17]; however, the effect of vitamin D supplementation on mortality might depend on the type of vitamin D (D2 or D3), the dose, the timing, the baseline serum level, and other factors [18,19].

The purpose of this study is to observe the effect of a high-calorie, high-in-protein oral nutritional supplement enriched with fructooligosaccharides (FOSs), calcium-β-hydroxy-β-methylbutyrate (CaHMB), and vitamin D (HC-HP-HMB-ONS) routinely used in clinical practice on nutritional status, recovery, the activity of daily living, quality of life, and length of hospital stay in older people with malnutrition or at risk of malnutrition after hip fracture surgery.

## 2. Subjects and Methods

This was an observational, prospective, national, multicenter study carried out in 17 hospitals in Spain between February 2020 and March 2022. This study was conducted in accordance with ethical principles that have their origins in the Declaration of Helsinki, the International Council for Harmonisation for Good Clinical Practice, and local laws and regulations. The protocol was approved by the Ethics Committee of Hospital La Paz (Madrid, Spain) on 31 January 2019 (Reference: PI-3503). The Ethics Committees of the 16 remaining hospitals were notified, following Spanish legislation.

The primary aim was to study the change in the subject’s nutritional status, measured by the Mini Nutritional Assessment (MNA) from baseline to week 12. Secondary assessments included study product compliance, the Short Physical Performance Battery (SPPB) score at week 12, and the change in the Barthel index for activities of daily living from baseline to week 12. As supportive endpoints, this study included the assessments of the body mass index, length of hospital stay, use of medication and supplements, healthcare-provider satisfaction, subject satisfaction, and laboratory test results.

### 2.1. Inclusion Criteria

Inclusion criteria were subjects 75 years of age or older who voluntarily signed the informed consent form, who were malnourished or at risk of malnutrition (determined with the MNA), being within three days post surgery for a hip fracture, with a glomerular filtration rate > 30 mL/min/1.73 m^2^ (MDRD equation estimated or measured), and who were under the care of a healthcare professional for malnutrition. The healthcare professional prescribed the study ONS to the patients (within the last 7 days prior to participating in this study). After surgery, the subjects went home, resided in a nursing home, or remained in hospital with an expected length of hospital stay below 15 days. The physicians who participated in this study were responsible for determining whether or not the subjects were suitable for participation in this study.

### 2.2. Exclusion Criteria

Subjects were excluded from this study if they had known severe dementia, brain metastases, or active cancer that, in the judgment of the study physician, would preclude study participation. In addition, subjects were excluded if they had eating disorders, a history of significant neurological or psychiatric disorders, or any other psychological conditions that could interfere with consumption of the study product. Additionally, those who did not have a caregiver who could assist them with adherence to the study protocol; had uncontrolled diabetes; were known to be allergic or intolerant to any ingredient found in the study product; or were participating in another study that required nutritional intervention were excluded from this study.

### 2.3. Nutritional Intervention

The oral nutritional supplement HC-HP-HMB-ONS (Ensure^®^ Plus Advance, Abbott Laboratories, S.A, Madrid, Spain) was selected because it contains a muscle-specific ingredient such as HMB among its ingredients. The dosage of the ONS administered was twice a day as a supplemental source of nutrition, according to the standard of care, for 12 weeks.

### 2.4. Study Variables

The Mini Nutritional Assessment is a 30-point scale screening tool used to identify adults who are malnourished or at risk of malnutrition. A score of <17 indicates malnourishment, 17 to 23.5 indicates a risk of malnutrition, and 24 to 30 indicates normal nutrition status [20].

The Barthel Index for Activities of Daily Living (ADL) is a scale that measures ten basic activities related to self-care and mobility: bowel control, bladder control, grooming, toilet use, feeding, transfers, mobility, dressing, climbing stairs, and bathing. The normal score is 100, and lower scores indicate greater dependency [21].

The SPPB includes three tests that assess static balance, gait speed, and lower limb strength. Each test is scored from 0 to 4, and the total score is 0–12 points [22]. A higher score indicates a higher functional status. In addition, the test is used in clinical practice to determine subjects’ frailty; thus, SPPB 0–6 is considered frail, SPPB 7–9 is considered pre-frail, and SPPB 10–12 is considered non-frail [23].

The healthcare satisfaction questionnaire contains 5 items (product tolerability, ability of the subject to meet their nutritional goals, nutritional status improvement, ease of use, and product recommendation) assessed using a 5-point Likert scale ranging from strongly agree to strongly disagree; one item (ability to achieve product recommendation) assessed using a 5-point evaluation scale (1 = 0%, 2 = 25%, 3 = 50%, 4 = 75%, and 5 = 100%); one item (dose reduction due to tolerability) assessed using a 3-point evaluation scale (1 = rarely, 2 = sometimes, and 3 = frequently); and one item (decision to continue with the product) assessed with a yes/no question [24]. The patient satisfaction questionnaire contains 4 items (taste, texture, tolerability, and general evaluation of the product) scored on a scale ranging from 1 to 10.

Demographic variables included age, sex, height, weight, primary and secondary diagnosis, time since hip fracture surgery, place of residence, and Charlson index.

Adverse events and serious adverse events reported by the subjects before and after the intake of the nutritional supplement were recorded and summarized. Continuous variables are presented as mean ± standard error of the mean (SEM). The Shapiro–Wilk test was used to check whether the measure deviated from a normal distribution. Categorical variables are presented by using frequencies and percentages. A significance level of 0.05 for all tests in contrast was used.

## 3. Results

A total of 310 subjects were included in this study; 40 were excluded due to not fulfilling the eligibility criteria. Therefore, 270 were considered suitable for analysis. The mean subject age was 87.3 years; 79.3% were women, 87.4% were living in a community, 30.1% were malnourished, and 69.9% were at risk of malnutrition. The mean subject BMI was 23.1 kg/m^2^. According to the Charlson index, 31.9% had high comorbidity, and 14.1% had low comorbidity; 97.8% of the subjects were taking one or more medications/supplements. The demographic and clinical characteristics of the subjects are shown in Table 1.

In terms of compliance, at the end of the 12 weeks of follow-up, overall, 82.1% of the subjects consumed more than 75% of the HC-HP-HMB-ONS prescribed; 196 subjects (60.7%) consumed 100% of the HC-HP-HMB-ONS prescribed; 42 (21.4%) consumed 75%; and 31 (15.8%) consumed 50%.

Seventy-seven subjects out of 270 discontinued the intake of HC-HP-HMB-ONS. Seventy-eight subjects did not complete the study and finished the study prematurely. The reasons for discontinuation are shown in Table 2.

**Figure 1:**
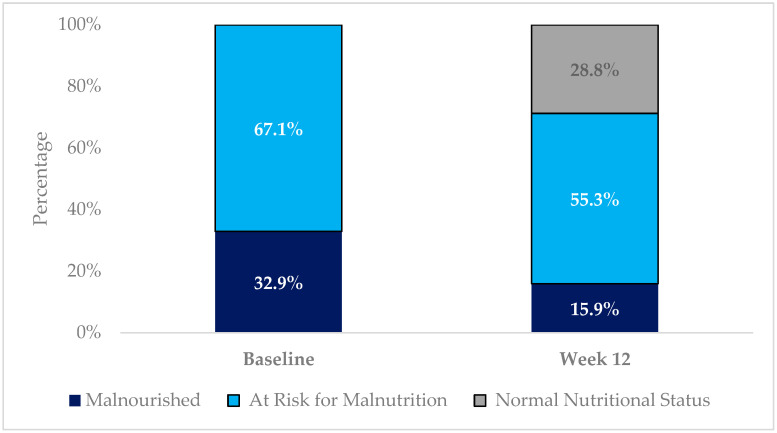
Changes in the subjects’ nutritional status from baseline to week 12, assessed with the Mini Nutritional Assessment tool.

At week 12, nutritional assessment was available in 171 subjects; of those, 50 (28.8%) had a normal nutritional status, 94 (55.3%) were at risk for malnutrition, and 27 (15.9%) were malnourished (Figure 1). The mean MNA total score showed a significant increase of 2.8 (*p* < 0.001) compared to baseline. Nutritional status improved in 46.8% of the subjects, was not modified in 48.2% of the subjects, and worsened in 5.3% of the subjects at week 12. Changes in the subjects’ nutritional status are shown in Table 3.

Regarding the supportive endpoints, subjects’ mean weight slightly increased by 0.310 kg at the end of the 12-week follow-up period compared to baseline, similarly to the body mass index; however, these increases were not statistically significant.

The Barthel ADL index significantly decreased from a mean of 68.2 ± 2.1 at baseline to 63.1 ± 1.9 at week 12 (*p* = 0.032) in the 177 subjects.

For the 170 subjects with the information available at the end of the 12-week follow-up, the mean Short Physical Performance Battery score at week 12 was 3.5 ± 0.2.

The mean length of the subjects’ hospital stay was 10.3 ± 0.3 days. Regarding concomitant medication, 264 (97.8%) subjects took some medication or supplements throughout the 12-week follow-up period. The mean score of the medications/supplements taken was 6.3 ± 0.2.

Biochemistry parameters associated with nutritional status improved significantly at the end of the 12-week follow-up, as shown in Table 4. The percentage of subjects with hemoglobin levels below 11 g/dL was reduced from 62.3% at baseline to 15.2% at week 12; mean hemoglobin levels increased from 10.48 ± 0.15 g/dL to 12.79 ± 0.28 g/dL. Hypovitaminosis D at baseline was present in 67.8% of the subjects, but this percentage dropped to 23% at the end of the 12 weeks of follow-up; mean plasma vitamin D levels increased from 16.4 ± 1.1 ng/mL to 36.5 ± 1.8 ng/mL (*p* < 0.001). In addition, the mean total protein level changed from 5.59 ± 0.6 g/dL to 6.6 ± 0.7 g/dL (*p* < 0.001), albumin changed from 3.2 ± 0.0 g/dL to 3.8 ± 0.0 g/dL (*p* < 0.001), and lymphocytes changed from 16.8 ± 0.9% to 27.0 ± 0.9% (*p* < 0.001).

**Figure 2:**
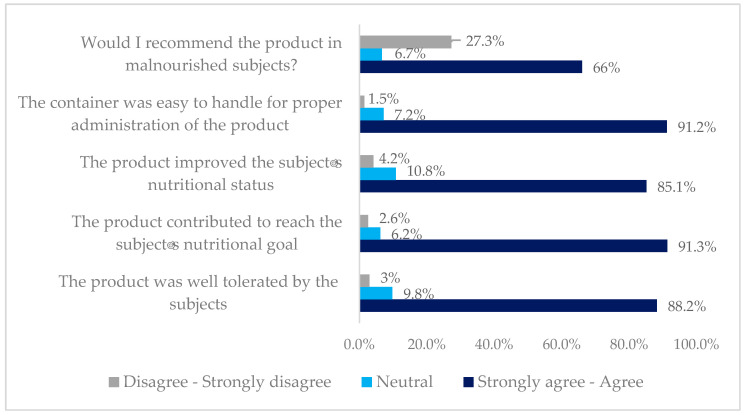
Assessment of the nutritional supplement by healthcare professionals.

In total, 87.1% of the healthcare professionals considered the HC-HP-HMB-ONS to be well tolerated, 91.3% stated that it helped subjects meet their nutritional goals, 85.1% stated that it led to an improvement in the subject’s nutritional status, and 66% stated that they would recommend HC-HP-HMB-ONS for malnourished subjects, but only 33.0% would recommend continuing with the product as the sole source of nutrition. Most of the healthcare professionals positively evaluated the use of HC-HP-HMB-ONS, and 77.8% of them “rarely” reduced the dose due to tolerability issues (Figure 2). At week 12, 193 subjects completed the Subject Satisfaction Questionnaire; HC-HP-HMB-ONS received a total score of 8.3 ± 0.1, scoring a mean of 8.1 ± 0.2 for taste, 8.2 ± 0.1 for texture, and 8.5 ± 0.1 for tolerability.

Throughout the 12-week follow-up, nine adverse events (all related to HC-HP-HMB-ONS) were reported, eight of mild intensity and one of moderate intensity; none of them were considered serious. Four AEs resulted in HC-HP-HMB-ONS discontinuation and led to a premature end of the study.

## 4. Discussion

Poor nutritional status and comorbidities prior to hip fractures, among others, are factors negatively associated with the health status, clinical outcomes, and quality of life of older patients after hip surgery [2,3,4,5,6,7,8,9,10,11,12,13,14,15,16,17,18,19,20,21,22,23,24,25,26,27,28,29]. Oral nutritional supplementation has been shown to be beneficial in preventing complications in older people after hip fractures [30,31,32,33,34]; consequently, the administration of nutritional supplements is recommended in this population [35].

The MNA is the tool most extensively used in clinical research, as well as in clinical practice, to assess the nutritional status of the older population [36]. Nutritional improvement, assessed with the MNA, has been associated with an improvement of the functional status of subjects with hip fractures at the time of hospital discharge, measured with the Functional Independence Measure (FIM) tool [37]. In addition, a meta-analysis published in 2019 revealed that malnutrition status, classified with the MNA, is associated with increased in-hospital mortality in older subjects with hip fractures treated surgically [38].

In our study, nutritional supplementation with HC-HP-HMB-ONS for 12 weeks improved the nutritional status of almost half of the subjects (46.8%), significantly increasing the mean MNA total score. In another similar study carried out in older people undergoing hip surgery, nutritional supplementation led to a significant increase in the mean MNA score in females, but the increase seen in males did not reach statistical significance [39]. In the study, the subjects received dietary council and two different types of oral nutritional supplements, one containing 7.5 g of protein and 150 calories/100 mL and another one containing 9.4 g of protein and 100 calories/100 mL, according to the results of individualized interviews. In this referenced study, the mean age of the subjects was 80 years, the proportion of females was 81%, and, regarding the subjects’ nutritional status, 3.1% were well nourished, 53.1% were at risk of malnutrition, and 43.7% were malnourished. Differences in the results obtained in this referenced study and our results might be explained by the differences between the two studies in the clinical/demographic characteristics of the subjects at baseline and the type of nutritional intervention performed.

Compliance with HC-HP-HMB-ONS differs according to studies. Thus, in a systematic review that included 46 studies in which compliance with ONS was analyzed, the average compliance was 78%, ranging from 37% to 100% [40]. Compliance with the ONS is an important aspect in the management of malnourished subjects, as poor adherence to ONS might put the goal of improving the nutritional status of the subjects at risk [41]. In our study, compliance with the prescribed HC-HP-HMB-ONS was high, with more than 80% of the subjects taking at least 75% of the prescribed nutritional supplement. This compliance was higher than the ones observed in a couple of studies in which a high-calorie and high-protein HMB-containing oral nutritional supplement was administered to old, malnourished subjects [42,43]. In one of these studies, throughout the 12-week study duration, 63% of the subjects took at least 75% of the ONS [42], and in the other one, throughout the 90-day study duration, approximately 33% of the subjects took at least 75% of the ONS [42].

Several biomarkers related to malnutrition in older people have been identified. Thus, low levels of vitamin D in serum have been associated with an increase in the risk of hip fracture in older patients [44]; however, vitamin D deficiency has not been associated with a higher risk of mortality in older people after hip fracture surgery [45]. In addition, vitamin D supplementation in this population has not been associated with a clinically significant improvement in their QoL [46]. On the contrary, low serum albumin levels have been associated with high mortality [38,47] and postoperative complications [48] in older patients after hip fracture surgery. An association has also been found between a low lymphocyte count [38], high neutrophil-to-lymphocyte ratio [49], and high platelet-to-lymphocyte ratio [50] with mortality in older patients after hip fractures [51]. In our study, after 12 weeks of nutritional supplementation with HC-HP-HMB-ONS, we found a significant improvement in albumin, lymphocyte count, and hemoglobin. Notably, the percentage of subjects with anemia was reduced from 62.3% at baseline to 15.2% at week 12, and this might be related to their nutritional status improvement.

We did not find a positive effect on the functional improvement of our subjects since the Barthel ADL index score decreased after 12 weeks of HC-HP-HMB-ONS administration. The decrease in the Barthel score, from 68.2 at baseline to 63.1 at week 12, was statistically significant, but according to the guidelines proposed for Barthel score interpretation, the subjects remained in the same dependency level throughout the study [52]. In this regard, Malafarina et al., in a randomized, controlled clinical trial, used the Barthel index to assess the daily living activities of older people with hip fractures admitted for rehabilitation therapy. The subjects were allocated to an intervention group or a control group; those in the intervention group received a standard diet plus HC-HP-HMB-ONS twice a day for 42.3 days on average, whereas subjects in the control group only received a standard diet. In this study, carried out in two rehabilitation centers, the Barthel index score changed from 95 prior to the hip fracture to 65 at the end of the treatment period. The recovery of daily living activities was more common in the subjects allocated to the nutritional supplementation group than in those allocated to the control group, but the difference was not statistically significant [53].

In our study, SPPB was assessed at the end of the 12-week treatment period. It was not possible to assess SPPB at baseline because the subjects were not able to ambulate at that time. The low mean SPPB score of 3.5 reflects the frailty of the participant subjects. This is important because a directly proportional relationship between SPPB scores and physical function in older adults has been found [54], as well as in subjects after hip surgery [55].

HC-HP-HMB-ONS was well tolerated by the subjects participating in our study. In total, seven subjects (2.6%) reported gastrointestinal adverse events related to the nutritional supplement. This is in line with the results of another study carried out with HC-HP-HMB-ONS in an older malnourished population in usual clinical practice in which 1.4% of the participants reported gastrointestinal adverse events [42]. In a randomized, placebo-controlled clinical trial in malnourished older hospitalized adults, the proportion of subjects assigned to the experimental treatment group (oral nutritional supplement containing high levels of protein and HMB) reporting gastrointestinal adverse events was higher (5% reported constipation, and 6% reported diarrhea). However, there was a similar proportion of subjects reporting gastrointestinal adverse events in the placebo group (5% reported constipation, and 6% reported diarrhea) [41].

In summary, HC-HP-HMB-ONS had a beneficial effect on the nutritional status of the remaining subjects and showed a good safety profile. The product was easy to use and both subjects and healthcare professionals showed a high level of satisfaction with this nutritional supplement. All of this can contribute positively to subjects’ adherence to treatment and thus make the recommendations of the European Society for Clinical Nutrition and Metabolism, regarding the administration of oral nutritional supplements in polymorbid subjects, easier to follow.

## 5. Limitations and Strengths

This study had some limitations, such as the lack of a control group and the fact that the body composition of the participating subjects was not analyzed. The muscle mass function measured by SPPB was assessed, but it was not possible to obtain the subjects’ SPPB score neither prior to the hip fracture nor at baseline. We would like to remark that during the recruitment period, the COVID-19 pandemic emerged, and it directly impacted the whole health system and led to 20% lower subject recruitment than that initially estimated. We consider the participation of 17 sites distributed throughout the Spanish territory and the recruitment of a significant number of subjects the strength of this study, as these provide us with a clear idea of the results that we can obtain with the use of HC-HP-HMB-ONS in clinical practice in this population.

## 6. Conclusions

The administration of HC-HP-HMB-ONS significantly improved the nutritional status of old and malnourished or at-risk-of-malnutrition subjects with hip fractures, measured according to the MNA, as well as the biochemical parameters associated with the subject’s nutritional status. There was a good compliance rate with HC-HP-HMB-ONS, which was well tolerated by most of the subjects. The subjects and healthcare providers expressed a high degree of satisfaction with HC-HP-HMB-ONS.

## Figures and Tables

**Table 1:** Clinical/demographic characteristics of the subjects at baseline.

Variable	No. of Subjects	
Age, years (mean ± SEM)	270	87.3 ± 0.3
Female sex, *n* (%)	270	214 (79.3)
Residential status, *n* (%)		
Community dwelling	270	236 (87.4)
Nursing home		34 (12.6)
Weight, kg (mean ± SEM)	266	58.1 ± 0.7
Height, cm (mean ± SEM)	267	158.4 ± 0.5
Body mass index, kg/m^2^ (mean ± SEM)	265	23.1 ± 0.2
Time from hip surgery to study baseline, days (mean ± SEM)	270	1.8 ± 0.1
Charlson Comorbidity Index (mean ± SEM)	270	2.0 ± 0.1
MNA score, (mean ± SEM)		
Screening score		8.4 ± 0.1
Assessment	270	10.1 ± 0.1
Total assessment		18.3 ± 0.1
Co-morbidities, *n* (%)		
Hypertension		203 (75.2)
Dyslipidemia		106 (39.3)
Smoker		13 (4.8)
Cardiovascular disease		127 (47)
Renal disease		31 (11.5)
Hepatobiliary disease		13 (4.8)
Cancer (current or past)		39 (14.4)
Diabetes	270	
Insulin-dependent		0 (0.0)
Not insulin-dependent		49 (18.1)
Other diseases		183 (67.8)
Barthel ADL index, (mean ± SEM)	177	68.2 ± 2.1
Anemia (hemoglobin < 11 g/dL), *n* (%)	138	86 (62.3)

SEM, standard error of the mean. MNA, Mini Nutritional Assessment. ADL, activities of daily living.

**Table 2:** Reasons for discontinuation.

Reasons for ONS-HC-HP-HMB Discontinuation, *n* (%)	
Investigator’s decision due to AEs	1 (0.4)
Subject’s decision due to AEs	4 (1.5)
Investigator’s decision for reasons other than AEs	4 (1.5)
Subject’s decision for reasons other than AEs	6 (2.2)
Non-compliance	32 (11.9)
Lost to follow-up	22 (8.1)
Other	8 (3.0)
Reasons for premature study discontinuation, *n* (%)	
Investigator’s decision due to AEs/change in medical status	5 (1.9)
Subject’s decision due to AEs/change in medical status	4 (1.5)
Subject’s decision for reasons other than AEs/change in medical status	5 (1.9)
Non-compliance	30 (11.1)
Lost to follow-up	22 (8.1)
Exitus	10 (3.7)
Other	2 (0.7)

ONS-HC-HP-HMB: High-calorie, high-in-protein oral nutritional supplement enriched with fructooligosaccharides (FOSs), calcium-β-hydroxy-β-methylbutyrate (CaHMB), and vitamin D.

**Table 3:** MNA change in status at baseline to week 12.

MNA Change in Status	*n* (%)
Improved	80 (46.8)
Maintained	82 (48.0)
Worsened	9 (5.3)

**Table 4:** Changes in laboratory parameters (mean values) from baseline to week 12.

Variable	*n*	Baseline	Week 12	Difference	*p*-Value
Total cholesterol (mg/dL)	137	137.2 ± 3.3	173.5 ± 3.8	36.34	<0.001
LDL-cholesterol (mg/dL)	95	76.2 ± 3.2	97.3 ± 3.6	21.07	<0.001
HDL-cholesterol (mg/dL)	100	46.6 ± 1.3	53.5 ± 1.4	6.93	<0.001
Triglycerides (mg/dL)	131	97.0 ± 3.7	112.3 ± 4.5	16.33	<0.001
Lymphocytes (%)	132	16.8 ± 0.9	27.0 ± 0.9	10.20	<0.001
Hemoglobin (g/dL)	144	10.48 ± 0.15	12.79 ± 0.28	2.31	<0.001
Total proteins (g/dL)	126	5.59 ± 0.6	6.65 ± 0.7	1.06	<0.001
Albumin (g/dL)	137	3.2 ± 0.03	3.82 ± 0.04	0.62	<0.001
Creatinine (mg/dL)	148	0.95 ± 0.03	0.85 ± 0.02	−0.10	<0.001
Glucose (mg/dL)	148	111.12 ± 3.41	100.30 ± 2.17	−10.82	<0.001
Vitamin D (ng/mL)	90	16.4 ± 1.1	36.5 ± 1.8	20.05	<0.001

## Data Availability

Data are contained within the article.
